# Active and Passive Surveillance and Phylogenetic Analysis of *Borrelia burgdorferi* Elucidate the Process of Lyme Disease Risk Emergence in Canada

**DOI:** 10.1289/ehp.0901766

**Published:** 2010-04-27

**Authors:** Nicholas H. Ogden, Catherine Bouchard, Klaus Kurtenbach, Gabriele Margos, L. Robbin Lindsay, Louise Trudel, Soulyvane Nguon, François Milord

**Affiliations:** 1 Centre for Food-Borne, Environmental and Zoonotic Infectious Diseases, Public Health Agency of Canada, Saint-Hyacinthe, Québec, Canada; 2 Groupe de recherche en épidémiologie des zoonoses et santé publique, Faculté de médecine vétérinaire, Université de Montréal, Québec, Canada; 3 Department of Biology and Biochemistry, University of Bath, Bath, United Kingdom; 4 Zoonoses and Special Pathogens Division, Public Health Agency of Canada, National Microbiology Laboratory, Winnipeg, Manitoba, Canada; 5 Laboratoire de santé publique du Québec, Institut national de santé publique du Québec, Québec, Canada; 6 Direction des risques biologiques et santé au travail, Institut national de santé publique du Québec, Québec, Canada

**Keywords:** *Borrelia burgdorferi*, climate change, emergence, environment, *Ixodes scapularis*, Lyme disease

## Abstract

**Background:**

Northward expansion of the tick *Ixodes scapularis* is driving Lyme disease (LD) emergence in Canada. Information on mechanisms involved is needed to enhance surveillance and identify where LD risk is emerging.

**Objectives:**

We used passive and active surveillance and phylogeographic analysis of *Borrelia burgdorferi* to investigate LD risk emergence in Quebec.

**Methods:**

In active surveillance, we collected ticks from the environment and from captured rodents. *B. burgdorferi* transmission was detected by serological analysis of rodents and by polymerase chain reaction assays of ticks. Spatiotemporal trends in passive surveillance data assisted interpretation of active surveillance. Multilocus sequence typing (MLST) of *B. burgdorferi* in ticks identified likely source locations of *B. burgdorferi*.

**Results:**

In active surveillance, we found *I. scapularis* at 55% of sites, and we were more likely to find them at sites with a warmer climate. *B. burgdorferi* was identified at 13 *I. scapularis*–positive sites, but infection prevalence in ticks and animal hosts was low. Low infection prevalence in ticks submitted in passive surveillance after 2004—from the tick-positive regions identified in active surveillance—coincided with an exponential increase in tick submissions during this time. MLST analysis suggested recent introduction of *B. burgdorferi* from the northeastern United States.

**Conclusions:**

These data are consistent with *I. scapularis* ticks dispersed from the United States by migratory birds, founding populations where the climate is warmest, and then establishment of *B. burgdorferi* from the United States several years after *I. scapularis* have established. These observations provide vital information for public health to minimize the impact of LD in Canada.

The Lyme disease (LD) epidemic, caused by the bacterium *Borrelia burgdorferi*, was first detected in North America in the late 1970s in association with expansion of populations of the tick *Ixodes scapularis* in northeastern and upper midwestern states of the United States (Spielman 1994). In the late 1980s, the one reproducing population of *I. scapularis* ticks known in Canada occurred at Long Point on the north shore of Lake Erie, but over the last decade more isolated populations of *I. scapularis* (and areas of endemic LD risk) became established (Ogden et al. 2009). Recent studies suggest that in southern Quebec *I. scapularis* is becoming established in wider regions rather than as isolated populations only (Ogden et al. 2008a), resembling the historical pattern seen in the northeastern United States. Migratory birds likely contribute to the northward dispersal of *I. scapularis* (Ogden et al. 2008b). However, we hypothesize that ticks carried northward can establish reproducing, self-sustaining populations, which pose the most significant risk of LD, only where climate (ambient temperature) conditions are suitable (Ogden et al. 2005, 2006a). “Adventitious” ticks dispersed by migratory birds from LD-endemic areas (where reproducing tick populations and *B. burgdorferi* transmission cycles are established) provide a low-level, geographically widespread LD risk in the Canadian environment and reduce the specificity of passive tick surveillance as a method of identifying LD-endemic areas (Ogden et al. 2006b). Deer and other terrestrial mammals may be important in dispersing *I. scapularis* over more local geographic ranges (Madhav et al. 2004).

*I. scapularis* has three developmental stages (or instars: larvae, nymphs, and adults) that all feed on woodland wild animal hosts. The ticks take a continuous blood meal on the same host for 3–10 days (depending on the instar). When fully engorged, the ticks fall off their host into the surface layers of the woodland floor and undergo development to the next instar. Ticks acquire *B. burgdorferi* infection while feeding on an infected host of a species capable of transmitting infection (i.e., a “competent reservoir” host). After molting, the ticks “quest” for another host among the herbage of the woodland floor, and infected ticks will infect any susceptible host they feed on (Spielman 1994).

Migratory birds and deer are probably key to *I. scapularis* dispersal, but how *B. burgdorferi* is dispersed is less clear. Some migratory bird species are competent reservoirs (Brinkerhoff et al. 2010), but infective northward-migrating birds are uncommon in spring, and northward migratory birds carry few *I. scapularis* larvae (which, if infected by the bird, would become infective nymphs that would feed on competent reservoir hosts) (Ogden et al. 2008b). Most *I. scapularis* carried north by migratory birds are nymphs (Ogden et al. 2008b), but these will molt into adults, which rarely contact competent reservoir hosts and feed mostly on reservoir- incompetent white-tailed deer (Telford et al. 1988). Therefore, immigration and establishment of *B. burgdorferi* in Canada may be a process that lags behind tick establishment.

In this study, we used active field surveillance to identify where LD-endemic areas are emerging at present, analyzed passive surveillance for ticks to provide data on recent history of *B. burgdorferi* emergence, and investigated genetic diversity of *B. burgdorferi* in ticks to understand potential sources of *B. burgdorferi* and *I. scapularis* in Quebec, Canada. Together, these data provide insight into the processes of *I. scapularis* and *B. burgdorferi* establishment, the emergence of *B. burgdorferi* in the Canadian environment, and the merit of these data for identifying emerging regions of endemic LD risk to inform the public health community.

## Materials and Methods

### Passive surveillance

Since 1990 *I. scapularis* ticks have been collected in Quebec province in a passive surveillance system involving voluntary participation of veterinary and medical clinics (Ogden et al. 2006b). Participating veterinary and medical doctors submit ticks to the Laboratoire de santé publique du Québec for identification. *I. scapularis* ticks are tested for *B. burgdorferi* infection at the National Microbiology Laboratory of the Public Health Agency of Canada. Data recorded included the instar, stage of engorgement (0 = unfed, 1 = semi-engorged, 2 = fully engorged), and host species (human, dog, or cat). Also recorded were residence locality of the person or animal on which the tick was found and whether or not they had, within 2 weeks before tick collection, traveled out of Quebec (in which case the corresponding data were not used in our analyses) or within Quebec (in which case the data were not used in cluster analysis).

From 1996 to 2004, ticks were analyzed for *B. burgdorferi* infection by a number of different polymerase chain reaction (PCR) methods, although these did not vary in their sensitivity and specificity (Ogden et al. 2006b). The most recently used of these methods (from 2003 to the present) comprises a two-test PCR procedure, as previously described [Ogden et al. 2006b, 2008b; see also Supplemental Material (doi:10.1289/ehp.0901766)].

In the present study we investigated space–time clustering of *B. burgdorferi*–infected ticks among ticks collected in passive surveillance from 1996 to 2008. Cluster analysis was performed in SaTScan version 8.0 (http://www.satscan.org/) using a Bernoulli model (Kulldorff 1997) with a temporal precision of 1 year for 1996–2008. Maximal spatial cluster size was set at 50% of the population; latitudes and longitudes for each submitted tick were obtained from Natural Resources Canada (2008) for the town or village of origin identified on the submission.

To ensure that any clusters discovered by this method were not explained by collinear space–time clustering of other variables, we investigated tick instar, stage of engorgement, host species (cat, dog, human, or other), and year of collection as explanatory variables and infection status of submitted *I. scapularis* ticks as the outcome variable in logistic regression models using Stata version 8.0 for Windows (StataCorp LP, College Station, TX, USA). Any significant variables were then compared against a binary variable “occurrence within or outside the cluster” in a multivariable logistic regression model constructed in Stata. Backward and forward substitution and elimination were used to obtain the most parsimonious multivariable model in which no variable could be removed without significantly affecting model deviance. We used *p* < 0.05 as the level of statistical significance throughout.

### Active surveillance

Field surveillance for the occurrence of established *I. scapularis* populations, and for evidence of *B. burgdorferi* transmission, was conducted at 71 woodland sites in three regions of southern Quebec (Montérégie, Montréal, and Estrie) during June through October of 2007 and 2008. Data on ticks collected at the 46 sites visited during 2007 have already been summarized (Ogden et al. 2008a). A further 25 sites were visited in 2008, and 13 sites where *I. scapularis* was found in 2007 were revisited in 2008. For information on site selection, see Supplemental Material (doi:10.1289/ehp.0901766). Dates of site visitation depended on permission from individual landowners.

At each visit, rodents were trapped and ticks were collected, and these were subsequently tested for evidence of *B. burgdorferi* infection [see Supplemental Material (doi:10.1289/ehp.0901766)]. Most sites were revisited once in October of 2007 or 2008, as part of another study to collect more questing adult ticks by flagging (dragging a cloth “flag” attached to a pole), but these data are included here. An ordinal tick population index was calculated for each site to give a value to the level of confidence that ticks found at a site came from a reproducing population, rather than being just bird-dispersed adventitious ticks (Ogden et al. 2006b, 2008a). The index was calculated based on the number of each of the three tick instars found at each site: 1 point if one tick was found, 2 points if 2–9 ticks were found, and 3 points if ≥ 10 ticks were found. Thus, the index had a minimum value of 0 (no *I. scapularis* found at the site) and a maximum of 9 (when ≥ 10 ticks of each of the three instars were found at the site). All questing ticks found in the environment and feeding ticks found on rodents were included in the calculation, but for statistical analyses we included only data collected at the first site visit.

To determine whether predicted temperature suitability for *I. scapularis* was associated with *I. scapularis* occurrence in the study, we investigated the tick population index as the outcome in ordinal logistic regression models (Long and Freese 2001). The explanatory variables included a value for the predicted temperature suitability (termed “predicted climate suitability”) for *I. scapularis* at the site, year of sampling, month of sampling (to account for seasonal variations in tick activity), and the number of rodents captured. The predicted climate suitability was the maximum tick abundance (a continuous variable) predicted by a simulation model for the mean annual cumulative degree-days (DD) > 0°C at each site. DD > 0°C, which captures temperature conditions on a multiyear scale relevant for tick population survival, was estimated for each site by interpolation of averaged meteorological station data (see Ogden et al. 2005, 2008a).

The location where *I. scapularis* populations establish could depend on the spatial structure of spread from existing populations in the United States or Quebec; therefore, we used robust estimates of errors in ordered logistic regression, so as not to assume independence of values for each site. To see how much spatial structure alone (rather than temperature or other variables) explained establishment of tick populations, we performed cluster analysis for an ordinal outcome in SaTScan using a Bernoulli model (Jung et al. 2007), in which the outcome was the tick population index. The spatial coordinates were obtained during site selection (Ogden et al. 2008a) and confirmed by global positioning system location at the time of the visit. We then investigated whether accounting for any observed clustering confounded associations between explanatory variables and the tick population index estimated by the multivariable ordinal regression analysis described above.

### Genetic diversity of *B. burgdorferi*

Multilocus sequence typing (MLST) was performed as previously described [Margos et al. 2008; see also Supplemental Material (doi:10.1289/ehp.0901766)] on 33 PCR-positive adult *I. scapularis* collected in Quebec in passive surveillance during 2005–2007 and on 7 PCR-positive questing adult *I. scapularis* collected in active surveillance by flagging the herbage at four of the field study sites (Figure 3). MLST is currently the most precise phylogeographic tool for *B. burgdorferi* (Hoen et al. 2009; Margos et al. 2008) and the best to identify whether *B. burgdorferi* in ticks in Quebec are a distinct, long-established population and, if not, the geographic origin of the *B. burgdorferi* found here. In addition, the 16S–23S intergenic spacer (IGS) locus and the gene encoding outer surface protein C (*ospC*) were amplified as described previously (Bunikis et al. 2004) and sequenced, because inferences regarding pathogenicity can be made from these sequences (Seinost et al. 1999).

## Results

### Passive surveillance

**Figure 1:**
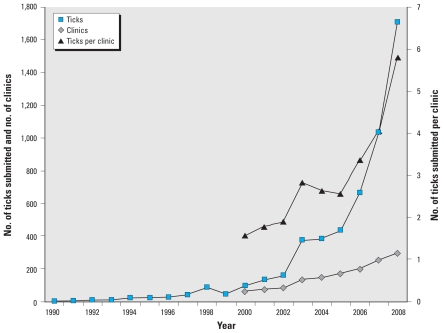
The number of *I. scapularis* ticks submitted in passive surveillance in Quebec, number of clinics (mostly veterinary practices) that participated in passive surveillance, and mean number of ticks submitted per clinic, by year.

An increase in the annual number of tick submissions up to 2003 may have resulted from increased participation in surveillance by veterinary clinics (Figure 1; Ogden et al. 2006b). However, after 2004, the numbers of ticks increased exponentially, to > 1,700 in 2008 [Figure 1; see also Supplemental Material, Table 1 (doi:10.1289/ehp.0901766)]. During this time, there was no marked increase in the numbers of participating clinics (Figure 1).

Before 2004, the mean prevalence of *B. burgdorferi* infection among adult ticks was 13.2% (128 of 984, Ogden et al. 2006b), but the mean prevalence of infection in ticks submitted from 2004 onward was lower (8.5%, 358 of 4,223). After excluding ticks collected from hosts that had recently traveled within or outside of Quebec and ticks for which data on tick instar or year of collection were missing, 3,222 ticks were eligible for the cluster analysis. At the time of analysis, full data were available only up to February 2008.

**Figure 2:**
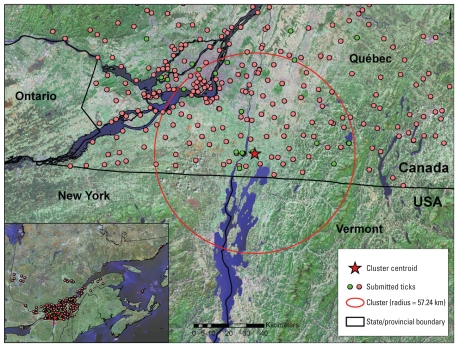
Locations from which ticks submitted in passive surveillance in Quebec during 1990–2008 were obtained. Also shown is the spatial extent of a cluster of ticks collected during 2004–2008 that had a low probability of being infected with *B. burgdorferi*. Green circles indicate locations where ticks were evaluated by MLST analysis.

Cluster analysis revealed one space–time cluster of ticks with significantly lower probability of being infected, located in the south of Quebec close to the U.S. border (with a radius of 57.24 km centered on 45.136° N, 73.192° W; Figure 2) and comprising ticks submitted during 2004–2008 (*p* < 0.001). The area corresponds to that in which we found possible established *I. scapularis* populations in active surveillance. In multivariable logistic regression models, the prevalence of infection within the space–time cluster remained significantly lower (4.9%) than the prevalence outside the cluster (12.9%) when accounting for host species and stage of engorgement [Table 1; see also Supplemental Material, Table 2 (doi:10.1289/ehp.0901766)]. Host species and stage of engorgement were collinear, with 56% of ticks from humans being unengorged, whereas only 3% and 4% of ticks from dogs and cats, respectively, were unengorged. The prevalence of infection in ticks within the space–time cluster was similar to the prevalence of infection in ticks submitted before 2004 from the whole region (13.2%). The total numbers of ticks submitted from within and outside the spatial limits of the cluster were 362 and 612, respectively, for the years before 2004. From 2004 onward, submissions from locations within the spatial limits of the cluster tripled (to 1,077), whereas submissions from outside this region doubled (to 1,168).

### Active surveillance

#### Ticks

**Figure 3:**
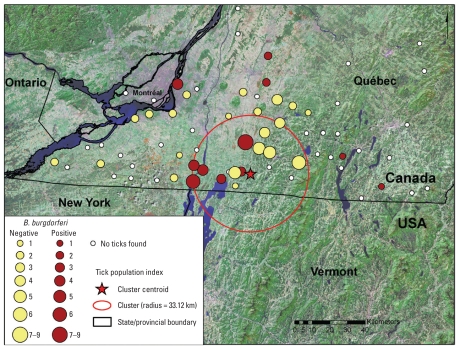
Study sites for active surveillance for *I. scapularis* establishment. In two cases, circles indicate the overlap of two populations. The size of the circles represents the tick population index for *I. scapularis* population establishment calculated from the number of ticks and the number of instars collected, as described in the text. Also shown is the spatial extent of a cluster of sites positive for *I. scapularis.*

On the first visit to the 71 sites, we found 574 *I. scapularis* at 35 sites [Figure 3; see also Supplemental Material, Table 3 (doi:10.1289/ehp.0901766)]. With additional flagging in October and revisits of some sites in 2008, *I. scapularis* were found at 39 of 71 (54.9%) of the sites and a total of 1,392 *I. scapularis* were collected. At all but one of the sites where *I. scapularis* were found in 2007, at least one tick was found when we revisited the sites in 2008. Overall, we found two instars at 14 sites and three instars at 10 sites within the same calendar year. A total 2,159 rodents were captured, and engorged ticks were collected from 293 of these rodents (13.6%; see Supplemental Material, Table 4).

In ordinal logistic regression analysis that accounted for variation between years, the tick population index was associated with sites with higher values for predicted climate suitability [Table 2; see also Supplemental Material, Figure 1 (doi:10.1289/ehp.0901766)]. The model output (Table 2) shows that for every one-point increase in the predicted climate suitability at a site, the odds that the tick population index at that site was in a higher category increased by 1.9%. For example, the odds that one or more ticks were present (i.e., that the tick population index was > 0) at the warmest site study (site 9, where the predicted climate suitability was 292, equivalent to a DD > 0°C of 3,495, and where the tick population index was 4) was 5.2 times greater than at the coolest site (site 32, where the predicted climate suitability was 17, equivalent to a DD > 0°C of 2,865, where no ticks were found and the tick population index was 0).

The number of rodents captured and the month of sampling were not significant predictors (*p* > 0.1 for both). We found a significant cluster of sites associated with the tick population index that centered on 45.11° N, 72.92° W with a radius of 33.12 km (*p* < 0.001; Figure 3). However, even though values for DD > 0°C at the sites are likely spatially autocorrelated, predicted climate suitability remained a significant determinant of the tick population index when the ordered logistic regression model was adjusted for the clustering (Table 2).

### *B. burgdorferi* infection in rodents and ticks

Evidence of transmission of *B. burgdorferi* (PCR-positive ticks or seropositive rodents) was found at 13 sites (Figure 3). Of the 1,169 *Peromyscus* spp., chipmunks, and squirrels that we tested serologically, 17 (1.45%) were positive for antibodies to *B. burgdorferi* [see Supplemental Material (doi:10.1289/ehp.0901766)]. The prevalence of infection in ticks was 1.8–3.3% (12–22 of 675; 11 ticks from one rodent were pooled) engorged larvae, 0.7% (1 of 135) questing nymphs, 9.9% (17 of 172) engorged nymphs, and 13.0% (19 of 146) questing adults [Supplemental Material, Table 4 (doi:10.1289/ehp.0901766)]. We did not test questing larvae because *B. burgdorferi* is not transmitted from female *I. scapularis* to their progeny (Patrican 1997). Excluding data from sites with no evidence of *B. burgdorferi* infection, 5.3% of rodents (17 of 318) were seropositive, and 2.3–4.1% of engorged larvae (12–22 of 533), 1% of questing nymphs (1 of 100), 12.9% of engorged nymphs (17 of 132), and 19% of questing adults (19 of 100) were positive for *B. burgdorferi* by PCR (see Supplemental Material, Table 4). Of the 19 positive questing adult *I. scapularis*, 11 were collected from three sites at which the combined prevalence was 26.2% (11 of 42). The mean prevalence of infection in adult ticks at all the other sites where we found *I. scapularis* was therefore 7.7% (8 of 104).

### Genetic diversity of *B. burgdorferi*

Of the 40 samples subject to MLST analysis, 7 (17.5%) showed mixed *B. burgdorferi* infections revealed by double peaks in the trace files. Samples with mixed infections and two samples with poor sequencing results were removed from further analysis. The 31 samples analyzed belonged to 15 sequence types (STs). All but one of the STs that were present in Quebec had previously been found in ticks collected in the United States: 28 in ticks collected in the Northeast and 2 previously found in the Midwest (Hoen et al. 2009). Although one sample (QC07-402) constituted a new ST not previously described from the United States, it is a single-locus variant of ST 36 carrying a point mutation in *nifS*. For details of the allelic profiles, see Supplemental Material, Table 5 (doi:10.1289/ehp.0901766).

Twenty-five percent of the samples (8 of 31) belonged to IGS type 1 and type 3, which correspond to restriction sequence type (RST) 1 that have been particularly associated with disseminated (i.e., severe) LD (Jones et al. 2006). Fifteen of the positive ticks carried *ospC* alleles (A, B, K, and I) that have also been implicated in disseminated LD (Seinost et al. 1999). One tick (QC07-484) carried *B. burgdorferi* with *ospC* allele L, an allele also found in ticks from migratory birds collected in Canada (Ogden et al. 2008b), whereas the ST of *B. burgdorferi* from this tick has previously been found only in ticks from the midwestern U.S. states (Illinois and Minnesota) (Hoen et al. 2009).

All four of the PCR-positive questing adults collected at one site [site 19; see Supplemental Material, Table 3 (doi:10.1289/ehp.0901766)] carried the same ST and were of the same IGS type, and three of the four carried the same *ospC* allele (Table 3).

## Discussion

Our findings suggest that the northern edge of emergence of *I. scapularis* populations and *B. burgdorferi* is currently in southern Quebec. This zone of emergence may be contiguous with endemic areas in neighboring regions of the United States (northern New York and Vermont) where *I. scapularis* populations and *B. burgdorferi* have been found (Diuk-Wasser et al. 2006).

*I. scapularis* ticks were found and submitted in passive surveillance from a geographic area of Canada that is much wider than that of known established *I. scapularis* populations (e.g., Ogden et al. 2006b). We have speculated that most ticks submitted in passive surveillance before 2004 were dispersed by migratory birds (Ogden et al. 2006b, 2008a). The prevalence of *B. burgdorferi* infection in these mostly adult ticks was 13.2%, similar to the prevalence of infection in engorged *I. scapularis* nymphs collected from migratory birds (15.4%; Ogden et al. 2008b). The number of ticks submitted in passive surveillance in Quebec increased each year from 2004 onward. Much of this increase was due to ticks submitted from a region near the U.S. border, and during the same period, *B. burgdorferi* infection prevalence in these ticks declined to 4.9%. Active surveillance showed that *I. scapularis* populations are becoming established in this region, although in many emerging tick populations *B. burgdorferi* either was not detected or occurred at low prevalence in questing adult ticks (7.7% at most sites). Together these findings are consistent with establishment of *I. scapularis* populations free of *B. burgdorferi* infection, resulting in an increase in the abundance of uninfected ticks in the environment that dilute the infection prevalence of adventitious ticks disseminated from the United States by migratory birds. This is reflected in an increase in ticks submitted in passive surveillance, with a decline in infection prevalence in these ticks. Therefore, passive surveillance may give an early signal of emergence of newly established *I. scapularis* populations and impending LD risk—declining infection prevalence combined with increasing numbers of submitted ticks.

Infection prevalence in ticks collected from dogs was lower than in those from cats [see Supplemental Material, Table 2 (doi:10.1289/ehp.0901766)], possibly due to some dogs being vaccinated with an anti-OspA vaccine clearing *B. burgdorferi* from preinfected ticks (Schwan and Piesman 2002). We found that unengorged ticks were less likely to be infected, consistent with multiplication of *B. burgdorferi* in infected ticks during engorgement (Schwan and Piesman 2002). This accounted for low infection prevalence in ticks from humans, which were mostly unengorged. These variations did not explain observed space–time clustering of uninfected ticks.

In the emerging *I. scapularis* populations we identified in active surveillance, the prevalence of infection in questing nymphal ticks was very low (1%) compared with other endemic sites in Canada, such as Long Point, Ontario (17%; Lindsay et al. 1997) and Lunenberg County, Nova Scotia (20%; Lindsay LR, unpublished data), and highly endemic areas of the northeastern United States (20–40%; Tsao et al. 2004). Nevertheless, at four sites, all three instars were found over 2 successive years, and *B. burgdorferi* was detected in the ticks, indicating that these sites are endemic areas for LD (Health Canada 1991). Two human LD cases have been associated with one site where all three tick instars and *B. burgdorferi*–positive ticks were found in 2008; analysis of *ospC* alleles and IGS types suggested that *B. burgdorferi* isolated from Quebec ticks is capable of causing disseminated LD (Jones et al. 2006). Together, these findings confirm the emergence of environmental risk for LD in southern Quebec.

In the present study, the spatial pattern of tick populations suggested that establishment may be partly due to local dispersal of ticks on terrestrial hosts rather than the presence of adventitious ticks carried into the region on migratory birds. Nevertheless, accounting for a lack of spatial independence of the sites, climatic conditions thought to particularly favor tick population survival (ambient temperature over the multiyear scale of the tick life cycle; Ogden et al. 2005) was a significant predictor of the occurrence of emerging populations. In the study design and analysis, we have attempted to control for habitat (in the selection of sites) and rodent host density, which are both likely to influence *I. scapularis* establishment. White-tailed deer densities in the study region are reportedly higher in the cooler eastern areas (hunting zone 5: 10.7 deer/km^2^) than in the warmer western region (hunting zone 8: 7.4 deer/km^2^) (Société de la Faune et des Parcs du Québec 2002). Therefore, we conclude that reproducing populations of *I. scapularis* are becoming established where the climate is warmer. This provides support for the possibility that *I. scapularis* populations and LD risk will increase at an accelerated rate with climate change (Ogden et al. 2008a) and that recent climate warming in Quebec (Bourque and Simonet 2008) could have facilitated the range expansion of this tick.

MLST typing suggested that *B. burgdorferi* in ticks collected in passive surveillance in Quebec are almost all identical to types of *B. burgdorferi* cultured from people with clinical LD and from questing ticks in the northeastern United States (Hoen et al. 2009; Margos et al. 2008). This finding provides the first firm support for the hypothesis that many *I. scapularis* ticks found in passive surveillance—and the *B. burgdorferi* infections they carry—are dispersed from the northeastern United States by migratory birds (Ogden et al. 2006a, 2008b). At one site, four questing adult ticks collected from different parts of the site all carried *B. burgdorferi* of an identical MLST type (already identified in the northeastern United States, Margos et al. 2008), and three of these ticks carried identical IGS types and the same *ospC* allele. Together, these findings support the hypothesis that transmission cycles of a *B. burgdorferi* ST from the northeastern United States are developing in a recently established *I. scapularis* population at this site.

## Conclusions

Active and passive surveillance for *I. scapularis* ticks and *B. burgdorferi* infection identified an emerging risk of LD in Quebec, possibly facilitated by a warming climate. MLST analysis of *B. burgdorferi* in ticks suggests that ticks and bacteria are most likely introduced from the northeastern United States, but surveillance data indicate that establishment of *B. burgdorferi* lags some years behind that of the tick population. Increasing numbers of ticks submitted in passive surveillance, as well as clusters of these ticks with low prevalence of infection, may provide an early signal of newly established tick populations that have not yet developed *B. burgdorferi* transmission cycles that are efficient enough to produce a high public health risk of LD. In Quebec and elsewhere in southeastern Canada, where *I. scapularis* are becoming established (Ogden et al. 2009), enhanced surveillance is needed to monitor how both *I. scapularis* ticks and *B. burgdorferi* infection are spreading in the environment.

## Figures and Tables

**Table 1:** Significant factors, in logistic regression models, associated with *B. burgdorferi* infection in ticks collected in passive surveillance.

Explanatory variable	No. positive/no. tested (%)^a^	OR (95% CI)	*p*-Value
Model A			
Ticks submitted from outside space–time cluster	273/2,139 (12.9)	Reference	
Ticks submitted from within cluster	57/1,083 (4.9)	0.38 (0.29–0.52)	< 0.001
Stage of engorgement			
No engorgement	15/253 (5.9)	Reference	
Semiengorged	250/2,394 (10.4)	1.77 (1.03–3.04)	0.038
Fully engorged	63/541 (11.6)	1.82 (1.01–3.27)	0.024

Model B			
Ticks submitted from outside space–time cluster	273/2,139 (12.9)	Reference	
Ticks submitted from within cluster	57/1,083 (4.9)	0.36 (0.27–0.49)	< 0.001
Host			
Human	15/280 (5.3)	Reference	
Dog	193/2,044 (9.4)	1.85 (1.08–3.20)	0.020
Cat	122/892 (13.7)	3.01 (1.72–5.26)	< 0.001

Abbreviations: CI, confidence interval; OR, odds ratio. Two models are presented because stage of engorgement and host of origin of ticks were collinear.

a Data on host or state of engorgement were missing from 20 ticks, so numbers of tested ticks are not identical for each variable.

**Table 2:** Significant variables in ordinal logistic regression models in which the outcome was the tick population index for each study site without (model A) and with (model B) accounting for whether or not sites occurred within an identified spatial cluster.

Explanatory variable	OR (95%CI)	*p*-Value
Model A		
Value for predicted climate suitability	1.019 (1.01–1.03)	< 0.001
2008 versus 2007	6.76 (1.79–25.53)	0.005

Model B		
Value for predicted climate suitability	1.017 (1.01–1.03)	0.001
2008 versus 2007	6.20 (1.66–23.10)	0.007
Site occurred within versus outside the cluster	10.80 (3.00–39.25)	< 0.001

Abbreviations: CI, confidence interval; OR, odds ratio.

**Table 3:** Results of analysis of MLST STs, IGS type, and *ospC* major group and the corresponding RST type for each sample analyzed.

Tick	ST	IGS type	*ospC* major group	RST	U.S. region^a^
QC07-785	1	1	A	1	NE
QC07-908	1	1	A	1	NE
QC07-161-5	1	1	A	1	NE
QC07-83	1	1	A	1	NE
QC07-493	3	2	K	2	NE
QC07-765	3	2	K	2	NE
QC07-84	3	2	K	2	NE
QC07-951	3	2	K	2	NE
QC07-1054	3	2	K	2	NE
QC07-565b	4	2	H	2	NE
QC07-755	7	3	B	1	NE
QC07-399	8	4	F	3	NE
QC07-603	8	4	F	3	NE
QC07-815	8	4	F	3	NE
QC07-819	8	4	F	3	NE
QC07-723	11	5	C	3	NE
QC07-776	12	6	M	3	MW
QC07-1008	14	6	G	3	NE
QC07-181-1^b^,^c^	14	6	G	3	NE
QC07-181-4^b^,^c^	14	6	G	3	NE
QC07-182-4^b^,^c^	14	6	G	3	NE
QC07-182-6^b^,^c^	14	6	A	3	NE
QC07-650	16	7	I	3	NE
QC07-484	29	2	L	2	MW
QC07-344	38	5	D	3	NE
QC07-362	34	5	J	3	NE
QC07-402^b^	238^d^	4	N	3	—
QC07-595	36	4	N	3	NE
QC07-851	59	3	B	1	NE
QC07-1048	59	3	B	1	NE
QC07-175-3	59	3	B	1	NE

a The region of the United States where each ST has been found: NE, Northeast; MW, Midwest.

b All ticks were engorged adult ticks collected in passive surveillance except these five questing adults collected in active surveillance.

c Data from these four questing adult ticks were collected at one field site during active surveillance.

d ST 238 is novel to this study.
